# Supplementary material for: The role of TRIF protein in regulating the proliferation and antigen presentation ability of myeloid dendritic cells through the ERK1/2 signaling pathway in chronic low-grade inflammation of intestinal mucosa mediated by flagellin-TLR5 complex signal
Source: PeerJ. 2024 Jan 2;12:e16716. doi: 10.7717/peerj.16716 (PMC10768658; doi:10.7717/peerj.16716)
Supplement: Supplemental Information 2 [file peerj-12-16716-s002.docx]

| **Table 1. MIQE checklist for authors, reviewers, and editors.a** |
| --- |
| **Item to check Importance Item to check Importance** |
| Experimental design qPCR oligonucleotides |
| Definition of experimental and control groups line 62-65、104 E Primer sequences 123-126 E |
| Number within each group 104 E RTPrimerDB identification number D |
| Assay carried out by the core or investigator’s laboratory? D Probe sequences Dd |
| Acknowledgment of authors’ contributions D Location and identity of any modifications none E |
| Sample Manufacturer of oligonucleotides D |
| Description 127 E Purification method D |
| Volume/mass of sample processed D qPCR protocol |
| Microdissection or macrodissection 123-126 E Complete reaction conditions 136-138 E |
| Processing procedure 127-132 E Reaction volume and amount of cDNA/DNA 133-136 E |
| If frozen, how and how quickly? no E Primer, (probe), Mg2+, and dNTP concentrations 123-128 E |
| If fixed, with what and how quickly? 130 E Polymerase identity and concentration 134-136 E |
| Sample storage conditions and duration (especially for FFPEb samples) 127 E Buffer/kit identity and manufacturer 75、80 E |
| Nucleic acid extraction Exact chemical composition of the buffer D |
| Procedure and/or instrumentation 138-140 E Additives (SYBR Green I, DMSO, and so forth) 80-82 E |
| Name of kit and details of any modifications none E Manufacturer of plates/tubes and catalog number D |
| Source of additional reagents used D Complete thermocycling parameters 152-153 E |
| Details of DNase or RNase treatment 130-133 E Reaction setup (manual/robotic) D |
| Contamination assessment (DNA or RNA) 138 E Manufacturer of qPCR instrument 75 E |
| Nucleic acid quantification 194-195 E qPCR validation |
| Instrument and method 129-140 E Evidence of optimization (from gradients) D |
| Purity (A260/A280) D Specificity (gel, sequence, melt, or digest) 130 E |
| Yield D For SYBR Green I, Cq of the NTC 131-132 E |
| RNA integrity: method/instrument 138 E Calibration curves with slope and y intercept 140 E |
| RIN/RQI or Cq of 3, and 5, transcripts 139-140 E PCR efficiency calculated from slope 140 E |
| Electrophoresis traces D CIs for PCR efficiency or SE D |
| Inhibition testing (Cq dilutions, spike, or other) 134 E r2 of calibration curve 140 E |
| Reverse transcription Linear dynamic range 140 E |
| Complete reaction conditions 136 E Cq variation at LOD 203 E |
| Amount of RNA and reaction volume 137 E CIs throughout range D |
| Priming oligonucleotide (if using GSP) and concentration 137-138 E Evidence for LOD 203 E |
| Reverse transcriptase and concentration 135 E If multiplex, efficiency and LOD of each assay 203 E |
| Temperature and time 139 E Data analysis |
| Manufacturer of reagents and catalogue numbers D qPCR analysis program (source, version) 138 E |
| Cqs with and without reverse transcription Dc Method of Cq determination 136-138 E |
| Storage conditions of cDNA D Outlier identification and disposition 138-139 E |
| qPCR target information Results for NTCs 139-140 E |
| Gene symbol 125 E Justification of number and choice of reference genes 125-126 E |
| Sequence accession number 126 E Description of normalization method 135-140 E |
| Location of amplicon D Number and concordance of biological replicates D |
| Amplicon length 132 E Number and stage (reverse transcription or qPCR) of technical replicates 139 E |
| In silico specificity screen (BLAST, and so on) 125 E Repeatability (intraassay variation) 139 E |
| Pseudogenes, retropseudogenes, or other homologs? D Reproducibility (interassay variation, CV) D |
| Sequence alignment D Power analysis D |
| Secondary structure analysis of amplicon D Statistical methods for results significance 182 E |
| Location of each primer by exon or intron (if applicable) none E Software (source, version) 75 E |
| What splice variants are targeted? 129 E Cq or raw data submission with RDML D |
| a All essential information (E) must be submitted with the manuscript. Desirable information (D) should be submitted if available. If primers are from RTPrimerDB, information on qPCR target, oligonucleotides, protocols, and validation is available from that source.  b FFPE, formalin-fixed, paraffin-embedded; RIN, RNA integrity number; RQI, RNA quality indicator; GSP, gene-specific priming; dNTP, deoxynucleoside triphosphate.  c Assessing the absence of DNA with a no–reverse transcription assay is essential when first extracting RNA. Once the sample has been validated as DNA free, inclusion of a no–reverse transcription control is desirable but no longer essential.  d Disclosure of the probe sequence is highly desirable and strongly encouraged; however, because not all vendors of commercial predesigned assays provide this  information, it cannot be an essential requirement. Use of such assays is discouraged. |
