# Supplementary figures and images for: The role of TRIF protein in regulating the proliferation and antigen presentation ability of myeloid dendritic cells through the ERK1/2 signaling pathway in chronic low-grade inflammation of intestinal mucosa mediated by flagellin-TLR5 complex signal
Source: PeerJ. 2024 Jan 2;12:e16716. doi: 10.7717/peerj.16716 (PMC10768658; doi:10.7717/peerj.16716)

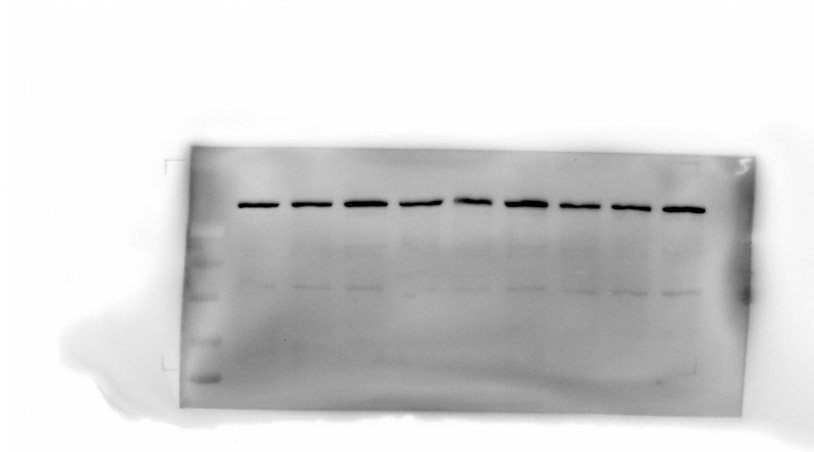

**TLR5**

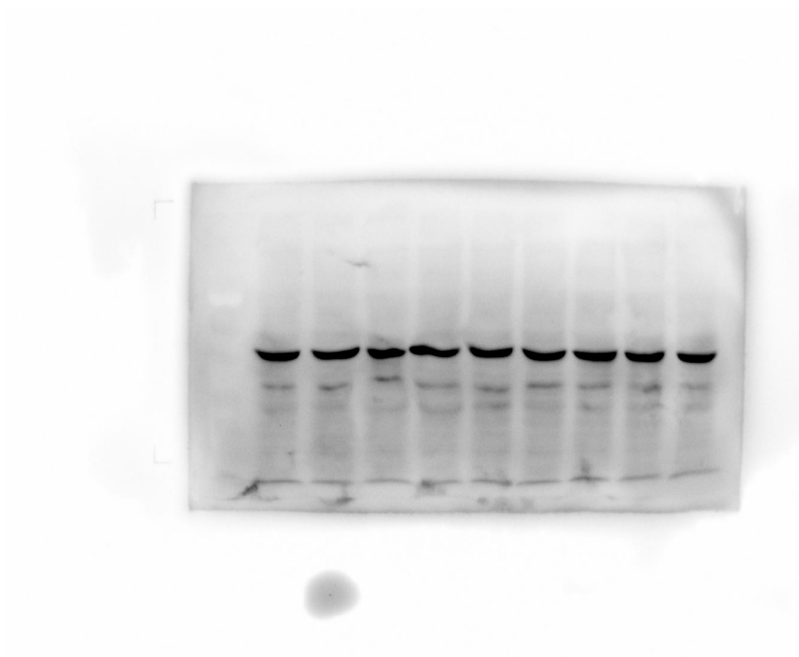

***$\beta$ -actin***

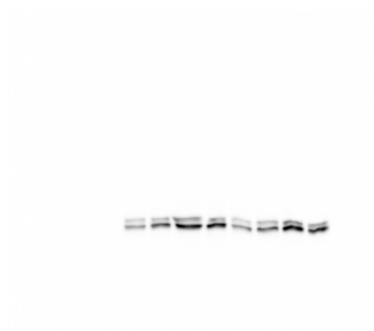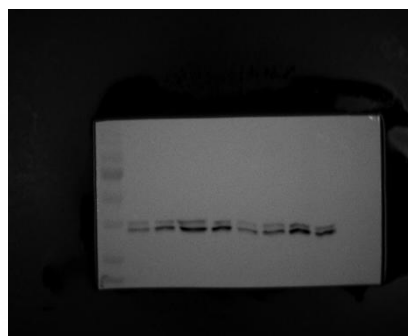

**pERK1/2**

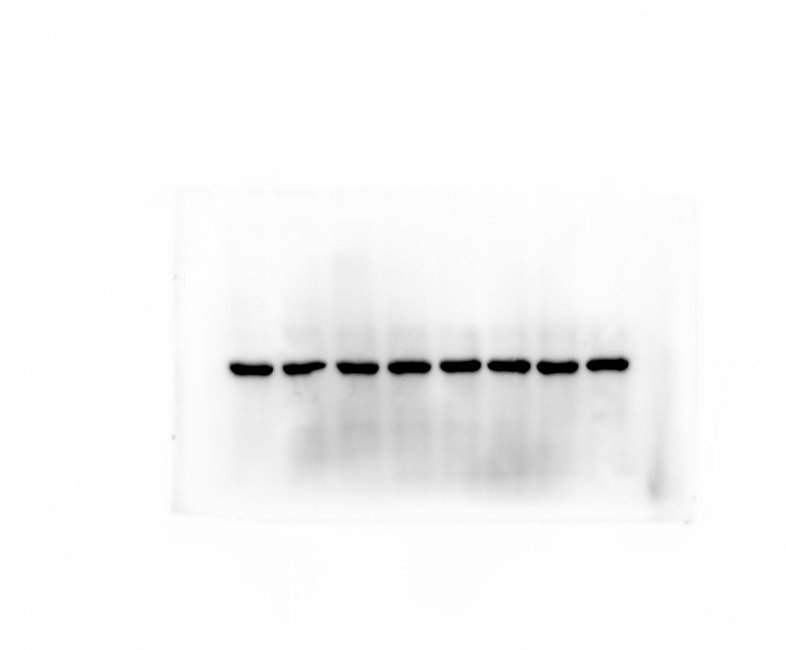

***β-actin***

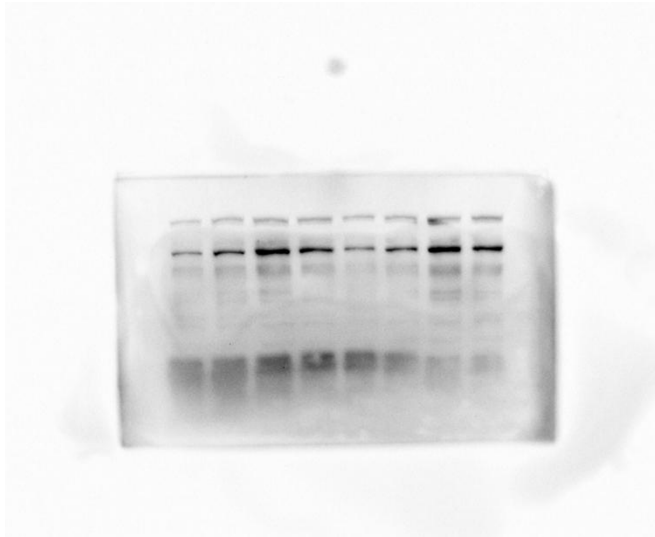

*TRIF*

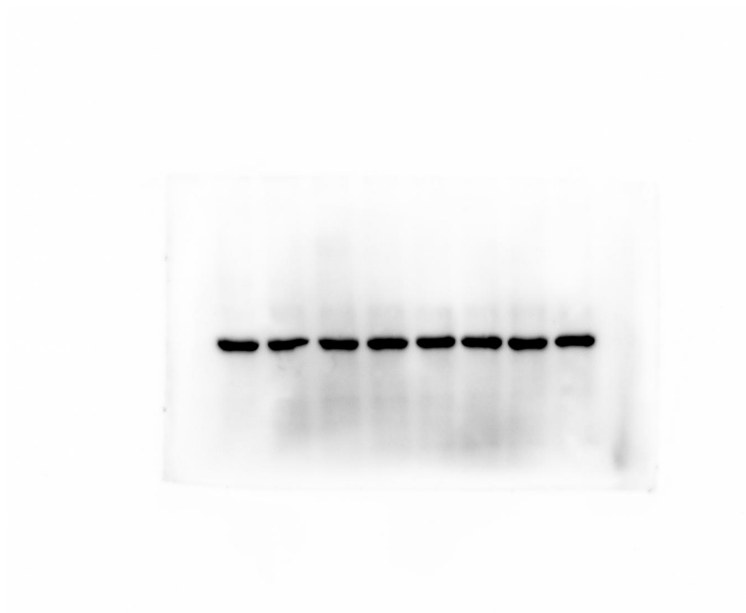

*$\beta$ -actin*

Supplement: Supplemental Information 3 [file peerj-12-16716-s003.pdf]
